# Hibernoma: a rare case of adipocytic tumor in head and neck.

**DOI:** 10.1186/s12901-017-0046-8

**Published:** 2017-12-15

**Authors:** Alexandra Rodriguez Ruiz, Sven Saussez, Thibaut Demaesschalck, Jérôme R. Lechien

**Affiliations:** 1 Department of Otolaryngology - Head and Neck Surgery, CHU Saint Pierre, Free University of Brussels, rue Haute 322, B1000 Brussels, Belgium; 2 Laboratory of Anatomy and Cell Biology, Faculty of Medicine, UMONS Research Institute for Health Sciences and Technology, University of Mons (UMons), Mons, Belgium

**Keywords:** hibernoma,, head,, neck,, tumor,, lipoma

## Abstract

**Background:**

Hibernoma is a rare soft tissue tumor stem from persistent fetal brown fat tissue. This benign tumor may occasionally occur in head and neck area and, in most cases, is characterized by an asymptomatic slow growth.

**Case presentation:**

We presented an uncommon case of hibernoma of the posterior cervical triangle occurring in a 30-year-old man referred to the department of otolaryngology. The patient suffered from a right, very painful, and rapidly growing mass since 3 months. MRI examination reported both an infiltrating mass and a homogenous enhancement of an underlying vascularization after the injection of intravenous contrast. According to the risk of sarcoma, a surgical procedure was made to completely excise the mass that was a hibernoma.

**Conclusions:**

Hibernoma may occur with an uncommon clinical presentation imitating malignancy. MRI plays a key role in the differential diagnosis and surgery remains the better therapeutic approach.

## Background

Hibernoma is a rare benign tumor originating from persistent fetal brown fat tissue [1]. The brown fat has a thermogenesis function, especially in the first years of a child’s life, but it regresses with age [1]. In adults, the most common residual areas of brown fat are usually located in the inter-scapular region, mediastinum, retroperitoneum, back, thigh and, sometimes, in head and neck [2–4]. Widely, the remaining of brown fat still remains asymptomatic and has no impact on the homeostasis. In rare cases, the remaining tissue can slowly grow, leading to the occurrence of a soft-tissue tumor. Thus, some cases of hibernoma are well described in the current literature and they are commonly found in chest, abdominal cavity and head and neck [3, 4]. In this paper, we reported an unusual case of hibernoma in a patient with a painless mass at the base of the neck. The current literature was reviewed about epidemiology, clinical course, diagnosis and treatment.

## Case presentation

**Fig. 1:**
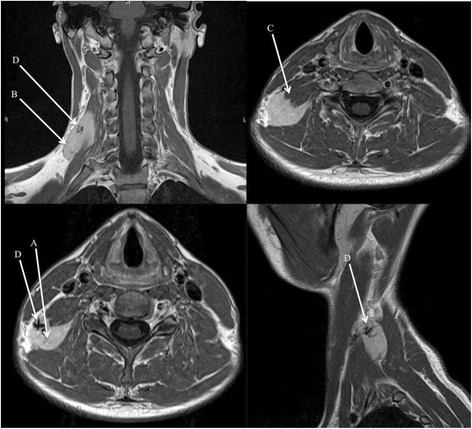
MRI of the hibernoma. The MRI (T1) revealed a 38 mm along the axis mass of the posterior cervical triangle with septa > 2 mm (**a**), nodular structures (**b**), muscular invasion (**c**), and a high vascularization (**d**).

**Fig. 2:**
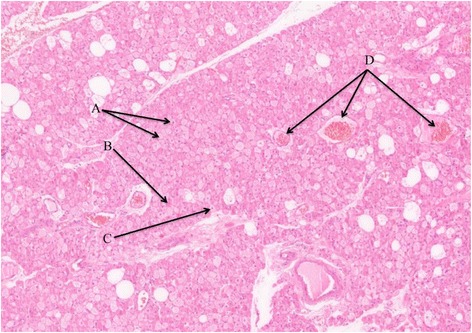
Histopathological findings. The histopathological findings (10×, hematoxylin & eosin) showed mature fat cells (**a**), abundant eosinophilic cells (**b**) with small cytoplasmic vacuoles and regular, small, round nuclei (**c**). The tissue was characterized by a high vascularization (**d**).

A 30-year-old man was referred to the Department of Otolaryngology and Head and Neck Surgery for mass located in the right posterior cervical triangle of the neck. The patient had this mass since several months but it recently started to grow in a context of substantial neck pain. The patient had no difficulty to breathe and swallow. Clinical examination exhibited a relatively mobile, soft mass located in the supraclavicular area. No cervical node was found. Both clinical and ultrasound examinations led to suspect a soft tissue mass, and the magnetic resonance imaging (MRI) revealed a 38 mm along the axis tumor (Fig. 1) between the elevator scapulae and the right scalene muscles. The tumor infiltrated the scalene muscles and the injection of intravenous contrast (gadolinium) reported a homogenous enhancement of an important underlying vascularization, a nodular structure of the tissue, and the presence of septa > 2 mm. According to the clinical features and the MRI characteristics (especially T1 sequence), we highly suspected liposarcoma of the neck. The fine needle aspiration biopsy was made but non-contributory. Thus, a surgical procedure was made to completely excise the mass and the macroscopic examination revealed an encapsulated taned-brown polylobulated tumor. The immediate post-operative follow-up was unremarkable. The definitive histopathological examination retained the diagnosis of a hibernoma, which was characterized by mature fat cells, abundant eosinophilic cells with small cytoplasmic vacuoles and regular, small, round cell nuclei (Fig. 2). The 4-years follow-up was unremarkable and the patient had no recurrence.

## Discussion

Since the first case described in 1906 [5], approximately ten cases of cervical hibernoma have been reported [6]. Among these, only three patients had hibernoma in the posterior cervical triangle but it seems highly probable that the diagnosis is widely underestimated [7, 8]. Indeed, with the contribution of modern technological advancements in positron emission tomography, some recent research supports the theory that the true prevalence of brown fat in adult is between 30 and 100%, suggesting an increased possibility to develop hibernoma, though often misdiagnosed or confused with lipoma [9]. Yet, it is important to make the difference between lipoma and hibernoma since, to date, no case of malignant transformation of hibernoma has been reported, which is not the case of lipoma. From an epidemiological standpoint, hibernoma is mostly seen in the third, fourth and fifth decades of life with a slightly higher female prevalence [6, 10].

Clinically, most patients with neck hibernoma are usually asymptomatic over time even if the slow growth of the tumor may, at some point, compress the adjacent structures [7, 8]. In our patient, the rapid growth and the related pain are uncommon manifestations and prompted us to quickly carry out additional examinations to exclude malignancy. To the best of our knowledge, only one reported case was characterized by similar clinical findings [6]. Among the complementary examinations, computed tomography (CT), MRI, and angiography can provide additional usefulness informations. So, hibernomas are usually depicted as well-circumscribed and variably homogeneous tumors with marked contrast enhancement. The differentiation with lipoma is possible with MR imaging because hibernoma still remains more vascularized, with large septa (>2 mm; easily seen with contrast agent), and, unlike to lipoma, the hibernoma tissue can be differentiated to the fat with MRI STIR or T2 Fat Sar sequences [11, 12]. Table 1 summarizes the clinical and imaging characteristics of lipoma, liposarcoma and hibernoma.

**Table 1:** Clinical and imaging characteristics of lipoma, liposarcoma and hibernoma.

Clinical features	Lipoma	Liposarcoma	Hibernoma	
Size	<5 cm (80% cases)	>5 cm	>5 cm	
Growth	Slow	Slow/moderate/high	Slow/moderate/high	
Age	25-65y	>50y	30-50y	
Clinic	Asymptomatic +++	Asymptomatic ++	Asymptomatic ++	
Sexe ratio	M = F	M > F	F > M	
MR Imaging	Well-homogeneous	Variably homogeneous	Variably homogeneous	
Characteristics	Well-circumscribed	Well-circumscribed	Well-circumscribed	
	Less vascularized	Variably vascularized (C+)	Variably vascularized (C+)	
	No nodular lesion	Possible nodular lesion	Possible nodular lesion	
	T1: Hyper (as fat)	T1: Hyper (less than fat/lipoma)	T1: Hyper (less than fat/lipoma)	
	T2: Hyper (as fat)	T2: Hyper	T2: Hyper	
	STIR/T2 Fat Sat: removing signal	STIR/T2 Fat Sat: no removing signal	STIR/T2 Fat Sat: no removing signal	
	Septa:<2 mm (C+)	Septa:>2 mm (C+)	Septa:>2 mm (C+)	

C + = contrast +; F = female; M = male; y = year. Many case presentations allowed the realization of this Table [2, 3, 6–8, 12, 13].

In our patient, the MRI examination showed a 38 mm along the axis, relatively well circumscribed tumor with intermediate signal intensity between subcutaneous fat and muscle. Moreover, the observed tumor was homogenous, relatively well circumscribed, with septa > 2 mm and some nodular areas. All of these features led us to exclude lipoma but the critical point concerned the differential diagnosis with liposarcoma. Indeed, the high vascularization, the large septa, and the fast arteriovenous contrast enhancement may mislead to liposarcoma diagnosis. Thus, as reported in the current literature, even with imaging, the characteristics of this tumor remain difficult to differentiate from malignant fat tumors and some very rare tumors such as angiolipoma and malignant fibrous histocytoma [6, 13, 14].

The final diagnosis is made after a fine needle aspiration procedure or after the surgical excision [6]. As showed in our patient, the histopathological findings include small, round, brown fat-like cells, variable numbers of mature fat cells with i) uniform, small eosinophilic cytoplasmic vacuoles, ii) regular, small, and round cell nuclei; and iii) delicate branching capillaries. Hypervascularization combined with abundant mitochondria give hibernomas their color. Concerning the histopathological differential diagnosis with liposarcoma, some cytology features (i.e. admixture of multivacuolated and univacuolated fat cells; a rich, delicate, capillary-like vasculature) are known to lead to a misdiagnosis of liposarcoma and the pathologists must take into consideration these similar characteristics. To date, hibernoma can be classified by morphologic or histological characteristics such as the presence of multivacuolated or univacuolated cells found in brown fat or normal fat [6]. Morphological, four variants of hibernoma are described: typical, myxoid, spindle cell, and lipoma-like [15]. Typical hibernoma included eosinophilic, pale, and mixed cell types. The myxoid variant contained a loose basophilic matrix while the spindle cell hibernoma had features of spindle cell lipoma. The lipoma-like variant only contained scattered cells. The present histopathological case corresponds to the typical variant. The 4-years follow-up of our patient did not report recurrence that seems to be in line with the other cases reported in the literature [6].

## Conclusion

Hibernoma is a rare benign tumor that can mimic malignant lesion of the soft tissue such as liposarcoma. In this paper, we report an unusual presentation of hibernoma of the posterior cervical triangle characterized by both severe pain and rapid growth. MRI plays a key role in the differential diagnosis, especially with other benign tumors but still remains limited for the differential diagnosis with malignancy. The biopsy and the surgery procedure correspond to the gold standard approaches for the final diagnosis, the exclusion of liposarcoma, and to select the appropriate treatment. To date, there is no described case of recurrence or malignant transformation.

## Abbreviations.

CT: Computed tomography
MRI: Magnetic resonance imaging.
